# Conservative treatment of acute traumatic posterior shoulder dislocations (Type A) is a viable option especially in patients with centred joint, low gamma angle, and middle or old age

**DOI:** 10.1007/s00167-022-06883-x

**Published:** 2022-01-29

**Authors:** Christian Festbaum, Marvin Minkus, Doruk Akgün, Andreas Hupperich, Dirk Maier, Alexander Auffarth, Marian Mitterer, Thomas Hoffelner, Mark Tauber, Lorenz Fritsch, Philipp Moroder

**Affiliations:** 1 Department of Shoulder and Elbow Surgery, Center for Musculoskeletal Surgery, Charité-Universitaetsmedizin Berlin, Campus Virchow, Augustenburger Platz 1, 13353 Berlin, Germany; 2 Department of Orthopaedic and Trauma Surgery, Faculty of Medicine, Medical Center, University of Freiburg, Freiburg im Breisgau, Germany; 3 Department of Orthopedics and Traumatology, Paracelsus Medical University Salzburg, Salzburg, Austria; 4 Department of Orthopedics, Herz-Jesu Krankenhaus Wien, Wien, Austria; 5 Department of Shoulder and Elbow Surgery, ATOS Clinic, Munich, Germany

**Keywords:** Posterior shoulder instability, Shoulder dislocation, Reverse Hill–Sachs lesion, Conservative therapy

## Abstract

**Purpose:**

Purpose of this study was to evaluate the mid- to long-term outcome after conservatively treated first-time posterior shoulder dislocations and to determine structural defects associated with failure.

**Methods:**

In this multi-centric retrospective study, 29 shoulders in 28 patients with first-time acute posterior shoulder dislocation (Type A1 or A2 according to the ABC classification) and available cross-sectional imaging were included. Outcome scores as well as radiological and magnetic resonance imaging were obtained at a mean follow-up of 8.3 ± 2.7 years (minimum: 5 years). The association of structural defects with redislocation, need for secondary surgery, and inferior clinical outcomes were analysed.

**Results:**

Redislocation occurred in six (21%) shoulders and nine shoulders (31%) underwent secondary surgery due to persistent symptoms. The posttraumatic posterior glenohumeral subluxation was higher in the redislocation group compared to the no redislocation group; however, statistical significance was not reached (61.9 ± 12.5% vs. 50.6 ± 6.4%). Furthermore, a higher adapted gamma angle was observed in the failed conservative treatment group versus the conservative treatment group, similarly without statistically significant difference (97.8° ± 7.2°, vs. 93.3° ± 9.7°). The adapted gamma angle was higher than 90° in all patients of failed conservative therapy and the redislocation group. An older age at the time of dislocation showed a significant correlation with better clinical outcomes (SSV: *r* = 0.543, *p* = 0.02; ROWE: *r* = 0.418, *p* = 0.035 and WOSI: *r* = 0.478, *p* = 0.045). Posterior glenohumeral subluxation after trauma correlated with a worse WOSI (*r* = − 0.59, *p* = 0.02) and follow-up posterior glenohumeral decentring (*r* = 0.68, *p* = 0.007). The gamma angle (*r* = 0.396, *p* = 0.039) and depth of the reverse Hill–Sachs lesion (*r* = 0.437, *p* = 0.023) correlated significantly with the grade of osteoarthritis at follow-up.

**Conclusion:**

Conservative treatment is a viable option in patients with an acute traumatic posterior shoulder dislocation with good outcome after mid- and long-term follow-up especially in patients with centred joint, low gamma angle, and middle or old age.

**Level of evidence:**

IV.

## Introduction

The term posterior shoulder instability (PSI) encompasses a large spectrum of different subpathologies. Determining the appropriate pathomechanism is a crucial step in the further management [8]. Recently, the ABC classification for PSI has been published, distinguishing acute (Type A), dynamic (Type B), and static (Type C) posterior shoulder instability and further subclassifying acute posterior shoulder instability into acute posterior subluxation (A1) and acute posterior dislocation (A2) [21]. The transition between patients with PSI Type A1 and A2 can be gradual and is characterised by increasing capsulolabral lesions and bony humeral and glenoid defects necessitating surgical treatment [21]. Although acute surgical treatment is warranted in patients with large and medially located reverse Hill–Sachs defects and large and displaced posterior glenoid rim fractures [19, 20], conservative treatment is a viable option for patients with only soft tissue or minor bony lesions after an acute posterior shoulder dislocation [21]. However, there is a lack of clinical and radiological outcome data after conservative treatment of posterior shoulder dislocations which would allow to determine critical structural defects and consequently the treatment type [33]. Therefore, the purpose of this study was to evaluate clinical and radiological mid- to long-term results of conservatively treated patients, who suffered an acute traumatic posterior shoulder dislocation. Furthermore, clinical and radiological risk factors related with inferior outcomes were assessed to provide a clinical guideline on which patients can be treated conservatively.

The hypothesis was that conservative treatment of acute traumatic posterior shoulder dislocations can lead to good clinical outcomes at mid- to long-term follow-up and that different structural defects of the joint are a risk factor for inferior outcome.

## Materials and methods

Approval from the institutional ethics committee (EA2/183/18) of the Charité University Hospital Berlin was obtained prior to onset of investigation.

### Patient selection

In this retrospective multi-centric study from the Arbeitsgemeinschaft für Arthroskopie und Gelenkchirurgie (AGA) data from four high-volume shoulder centres were collected.

A database research was initially carried out in each centre to identify patients treated for acute first-time posterior shoulder dislocations between 2003 and 2014. Inclusion criteria were (1) a type A1 or A2 posterior shoulder instability according to the ABC classification of PSI [21], (2) age > 18 years, (3) minimum follow-up of 5 years since the first posterior shoulder instability event, (4) an initial conservative treatment strategy and (5) presence of a Computerised Tomography (CT) or Magnetic Resonance Imaging (MRI) scan of the affected shoulder at the time point of the trauma.

Excluded were all patients that (1) sustained a humeral head fracture dislocation (except for reverse Hill–Sachs lesions) (2) were not reduced and remained in a chronic locked position, and (3) suffered from a bidirectional shoulder instability, or (4) died during follow-up period.

Forty-five shoulders in 44 patients met our inclusion and exclusion criteria. Sixteen patients could not be contacted due to missing contact information or refused to participate in this study, so a total of 29 shoulders in 28 patients were available for final follow-up examination (64%).

### Conservative treatment

Due to the multi-centric design of the study, the conservative treatment strategies slightly varied between patients. However, shoulders of all patients were immobilised in an abduction pillow or neutral rotation brace for 2–6 weeks. Physiotherapy was conducted for 2–27 weeks.

### Patient characteristics

Mean follow-up was 8.3 ± 2.7 years (range 5–14.3 years). Mean age at time of first episode was 40 ± 13.7 years (range 18–75 years). Twenty-three patients were male (82%). Dominant side was affected in 13 cases (45%) and non-dominant side in 16 cases (55%). One patient suffered from a posterior dislocation of both shoulders. Regarding the ABC classification, 4 shoulders (14%) suffered an A1 and 25 (86%) an A2 PSI. The cause of the dislocation was a fall in 24 shoulders (83%) including falls with the bicycle in 15 shoulders. A convulsive episode (1 electric accident and 1 epileptic seizure) was the reason for dislocation in two shoulders. A car accident was the cause for dislocation in two and a gymnastics injury in one shoulder.

### Clinical investigation

At follow-up, patients were examined and the following parameters were recorded: history of the affected joint, range of motion (ROM), Beighton-Score [4], and clinical outcome and activity scores including the Rowe Score [27], the Western Ontario Shoulder Instability (WOSI) Index [14], the Subjective Shoulder Value (SSV) [11] and the Shoulder Sports Activity Score (SSAS) [30].

### Radiological investigation

**Fig. 1:**
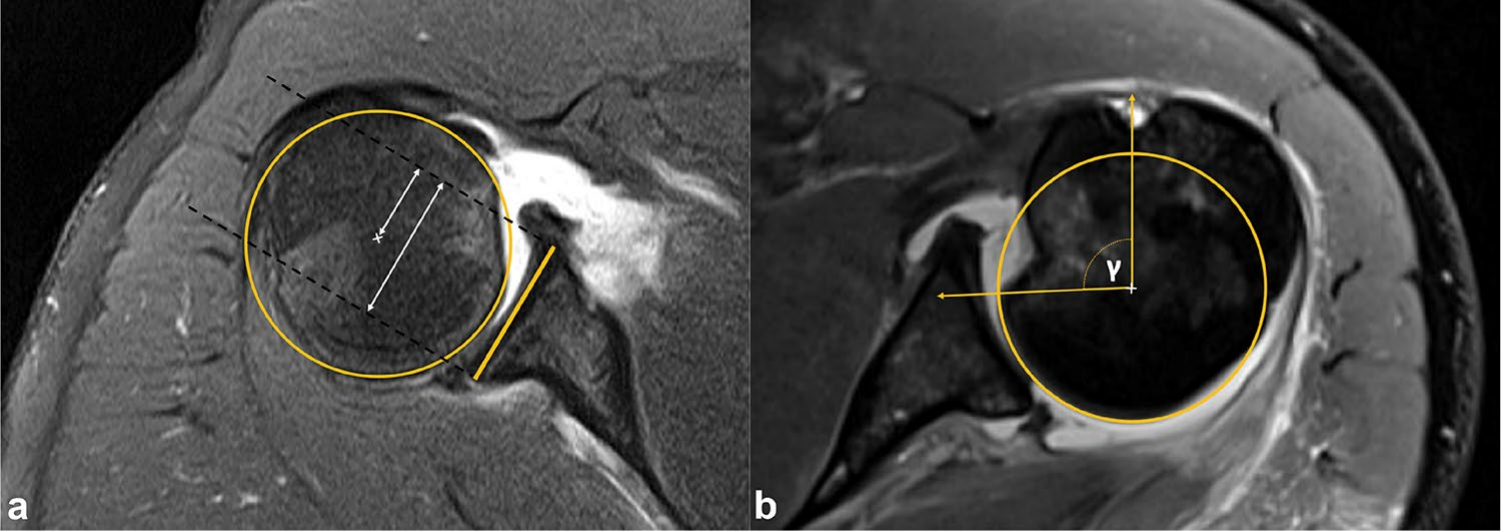
Radiological measuring of the glenohumeral centring and gamma angle of the RHSL. **a** To determine the centring of the humeral head in relation to the glenoid, a best-fit circle was placed on the remainder of the intact humeral articulating surface. A tangential line was drawn on the bony glenoid width, with two perpendicular lines starting from the anterior and posterior glenoid rims (dashed lines). Distances from the centre of the circle to the anterior dashed line was measured in relation to the distance from the anterior to the posterior dashed line and expressed as a percentage according to a previously published technique [1, 32]. Therefore, values > 50% represent a posterior glenohumeral decentring and values < 50% an anterior decentring, respectively. In this figure, the posterior glenohumeral decentring value is 21.8/38.3 = 56.9%. **b** Similar to the measurement of the glenohumeral centring, a best-fit circle was placed on the humeral head and lines were drawn from the posterior edge of the reverse Hill–Sachs defect to the centre of the circle and from the bicipital sulcus to the centre of the circle. The angle between both lines is the gamma angle which provides information on the size and localisation of the RHSL

Conventional radiographs of the involved shoulder were performed in 24 (86%) patients at final follow-up examination. Presence and progression of instability arthropathy was evaluated according to Samilson and Prieto in posttraumatic and in final follow-up radiographs [28]. A follow-up MRI scan was available in 22 shoulders (76%). The reverse Hill–Sachs lesion (RHSL), glenoid defect, glenohumeral and scapulohumeral centring and glenoid version were assessed on posttraumatic and follow-up cross-sectional imaging as follows:

RHSLs were measured on axial CT or MRI tomographic images displaying the greatest extent of the defect using the alpha, beta, and gamma angles as previously published and proven reliable (Fig. 1B) [20, 22]. As RHSLs are oriented parallel to the humeral shaft, axial images are best suitable to measure the extent of the RHSL [19]. Moreover, it has been shown that MRI and CT images render comparable measurement results [23]. Glenohumeral centring was measured as previously published by Walch et al. and illustrated in Fig. 1A [1, 32]. Scapulohumeral centring was measured according to Kidder et al. [13]. Glenoid version was measured according to the technique published by Friedman et al. [9]. Glenoid defects were measured according the Pico-method to calculate the percentage of bone loss [5]. The size/diameter of the posterior glenoid rim lesion was measured according to Baudi et al. [3]. An adapted gamma angle was calculated by adding 2.3° per millimetre of glenoid defect to the gamma angle measurement [19].

The radiological assessment was independently performed twice by two raters (M.M. and C.F.) with at least 5 weeks between both measurements.

All measurements were performed with Visage 7.1 (Visage Imaging, Berlin, Germany) (Fig. 1).

### Assessment of shoulder arthropathy

To enable easier comparability of the long-term shoulder arthropathy, the collective instability arthropathy (CIA) index was used to quantify the degree of osteoarthritis. The CIA-Index is calculated by assigning a corresponding number of points between 0 and 3 for each instability arthropathy grade according to the classification of Samilson and Prieto [28]. Subsequently, the sum of all calculated points is then divided by the total number of examined patients, respectively, shoulders. The index, therefore, ranges from 0, meaning none of the patients showed a sign of instability arthropathy, to 3, meaning all patients featured a grade 3 instability arthropathy [18].

### Statistical analysis

Inter- and intraclass correlation coefficient (ICC) with a 95% confidence interval (CI) was calculated for all measurements. As recommended by Portney et al., an ICC < 0.75 indicates moderate reliability, 0.75–0.90 good reliability, and an ICC > 0.9 indicates excellent reliability for clinical measures [26]. After reliability assessment, values of both raters were averaged for further analysis.

The patients were separated into groups depending on the success of the initial conservative therapy (conservative therapy vs. failed conservative therapy) and event of a redislocation (redislocation vs. no redislocation). A failed conservative therapy was defined as a surgical intervention during the follow-up time.

The Kolmogorov–Smirnov test was used to test for normal distribution. The two-sample *t*-test (for parametric distribution) or Mann–Whitney *U* test (for nonparametric distribution) was used to compare continuous variables between groups. Correlations between the patient-specific characteristics like age at time of dislocation, defect characteristics and glenohumeral centring and clinical outcome were calculated using the correlation coefficients of Pearson (for parametric distributed variables) and Kendall and Spearman Rank Correlation (for nonparametric distributed variables). To determine the strength of association between categorical variables and interval level variables, the eta coefficient was calculated.

## Results

Inter- and intrarater reliability for all measurement parameters is displayed in Table 1.

**Table 1:** Interrater reliability displayed in terms of intraclass correlation coefficient (ICC)

	ICC (95% CI)	According to Portney et al.
Measurement parameter		
Posttraumatic alpha angle	0.83 (0.65–0.92)	Good reliability
Posttraumatic beta angle	0.92 (0.84–0.96)	Excellent reliability
Posttraumatic adapted gamma angle	0.88 (0.75–0.94)	Good reliability
Posttraumatic depth of RHSL	0.96 (0.90–0.98)	Excellent reliability
Posttraumatic glenoid defect area	0.98 (0.96–0.99)	Excellent reliability
Posttraumatic glenohumeral centring	0.89 (0.76–0.95)	Good reliability
Posttraumatic glenoid version	0.96 (0.92–0.98)	Excellent reliability
Posttraumatic scapulohumeral centring	0.91 (0.83–0.94)	Excellent reliability
Follow-up glenohumeral centring	0.87 (0.69–0.95)	Good reliability
Follow-up osteoarthritis	1 (1–1)	Excellent reliability

*CI* confidence interval, *RHSL* reverse Hill–Sachs lesion

Redislocation occurred in six (21%) shoulders and nine shoulders (31%) had to undergo surgery after failed conservative treatment due to persistent symptoms. Inferior clinical outcomes were noted at the time of final follow-up for the patients with failed conservative therapy. The failed conservative therapy group had a significantly lower SSV compared to the conservative group (80 ± 21.9 vs. 93.2 ± 8.1, *p* = 0.041). No significant differences were found in clinical outcomes between the redislocation and the no redislocation subgroup (Table 2).

**Table 2:** Clinical scores at final follow-up

	Conservative therapy (*N* = 20)	Failed conservative therapy (*N* = 9)	*p*-value	No redislocation (*N* = 23)	Redislocation (*N* = 6)	*p*-value
Outcome						
SSV, mean ± SD	93.2 ± 8.1	80 ± 21.9	0.04	92.8 ± 7.6	75 ± 26.8	n.s.
ROWE, mean ± SD	96.1 ± 10.2	82.5 ± 33.4	n.s.	96.7 ± 9.5	72 ± 39.8	n.s.
WOSI Score, mean ± SD	88.2 ± 13.1	78.5 ± 20.5	n.s.	88.2 ± 12.3	72.6 ± 24.4	n.s.
Flexion, mean ± SD, °	178 ± 4	179 ± 4	n.s.	178 ± 4	176 ± 5	n.s.
Abduction, mean ± SD, °	177 ± 5	177 ± 8	n.s.	177 ± 5	175 ± 10	n.s.
Glenohumeral abduction, mean ± SD, °	97 ± 8	91 ± 27	n.s.	97 ± 8	86 ± 38	n.s.
External rotation, mean ± SD, °	64 ± 5	61 ± 19	n.s.	65 ± 15	55 ± 17	n.s.
Internal rotation, median	Thoracic vertebrae 12	Thoracic vertebrae 12	n.s.	Thoracic vertebrae 12	Thoracic vertebrae 12	n.s.

*SSV* subjective shoulder value, *WOSI* Western Ontario shoulder instability

Comparison of patients’ clinical characteristics and radiological measurements between groups are displayed in Table 3.

**Table 3:** Comparison of subgroup characteristics

	Conservative therapy (*N* = 20)	Failed conservative therapy (*N* = 9)	*p*-value	No redislocation (*N* = 23)	Redislocation (*N* = 6)	*p*-value
Patient characteristics						
Age at time of initial dislocation	42 ± 11.4	36.3 ± 18	n.s.	41.4 ± 11.2	35.8 ± 21.7	n.s.
Follow-up, mean ± SD, months	106.1 ± 33.2	81.9 ± 23.6	n.s.	100.6 ± 34.0	90.7 ± 24.6	n.s.
Sex						
Male, *N* (%)	18 (90)	6 (67)	n.s.	20 (87)	4 (67)	n.s.
Female, *N* (%)	2 (10)	3 (33)		3 (13)	2 (33)	
Affected side						
Right side, *N* (%)	7 (35)	3 (33)	n.s.	7 (30)	3 (50)	n.s.
Left side, *N* (%)	11 (55)	6 (67)		14 (61)	3 (50)	
Both sides, *N* (%)	1 (10)	0 (0)		1 (9)	0 (0)	
Dominant side, *N* (%)	10 (50)	3 (33)	n.s.	10 (44)	3 (50)	n.s.
Non-dominant side, *N* (%)	10 (50)	6 (67)		13 (56)	3 (50)	
Beighton-Score, mean ± SD	1.2 ± 1.2	1.4 ± 1.9	n.s.	1.3 ± 1.4	1 ± 1.4	n.s.
SSA-Score, mean ± SD	6.2 ± 2.1	6.3 ± 2.9	n.s.	6 ± 2.2	7 ± 3	n.s.
Posttraumatic radiological parameters						
Alpha angle, mean ± SD	43.3 ± 5.9	46.1 ± 8.6	n.s.	43.8 ± 6.8	45.6 ± 7.4	n.s.
Beta angle, mean ± SD	48.4 ± 8.6	47.3 ± 11.1	n.s.	48.5 ± 8.7	46.3 ± 12.1	n.s.
Gamma angle, mean ± SD	91.7 ± 9.5	93.4 ± 5.9	n.s.	92.3 ± 9	91.9 ± 6.6	n.s.
Adapted gamma angle, mean ± SD	93.3 ± 9.7	97,8 ± 7,2	n.s.	94.4 ± 10.2	96.0 ± 2.9	n.s.
Depth of RHSL, mean ± SD, %	15.2 ± 5.3	11.2 ± 2.3	0.01	14.6 ± 5.2	11.3 ± 2.5	n.s.
Glenoid defect area, mean-area ± SD, %	1.6 ± 1.9	4.1 ± 6.6	n.s.	1.9 ± 2.9	4 ± 7.1	n.s.
Glenoid defect diameter, mean-diameter ± SD, mm	0.7 ± 1.5	1.9 ± 2.8	n.s.	0.9 ± 1.9	1.8 ± 2.6	n.s.
Glenohumeral centring, mean ± SD, %	50.6 ± 6.6	58.2 ± 11.8	n.s.	50.6 ± 6.4	61.9 ± 12.5	n.s.
Glenoid version, mean ± SD	8.2 ± 3.6	8.7 ± 2.8	n.s.	8.2 ± 3.4	8.8 ± 3.4	n.s.
Scapulohumeral centring, mean ± SD, %	58.8 ± 6.0	63.0 ± 10.9	n.s.	59.3 ± 6.3	63.4 ± 12.7	n.s.

*SSA-Score* subjective shoulder activity-score, *RHSL* reverse Hill–Sachs lesion

The posttraumatic posterior glenohumeral subluxation was higher in the redislocation group compared to the no redislocation group; however, statistical significance was not reached (61.9 ± 12.5% vs. 50.6 ± 6.4%). Furthermore, a higher adapted gamma angle was observed in the failed conservative treatment group versus the conservative treatment group, similarly without statistically significant difference (97.8° ± 7.2°, vs. 93.3° ± 9.7°).

**Fig. 2:**
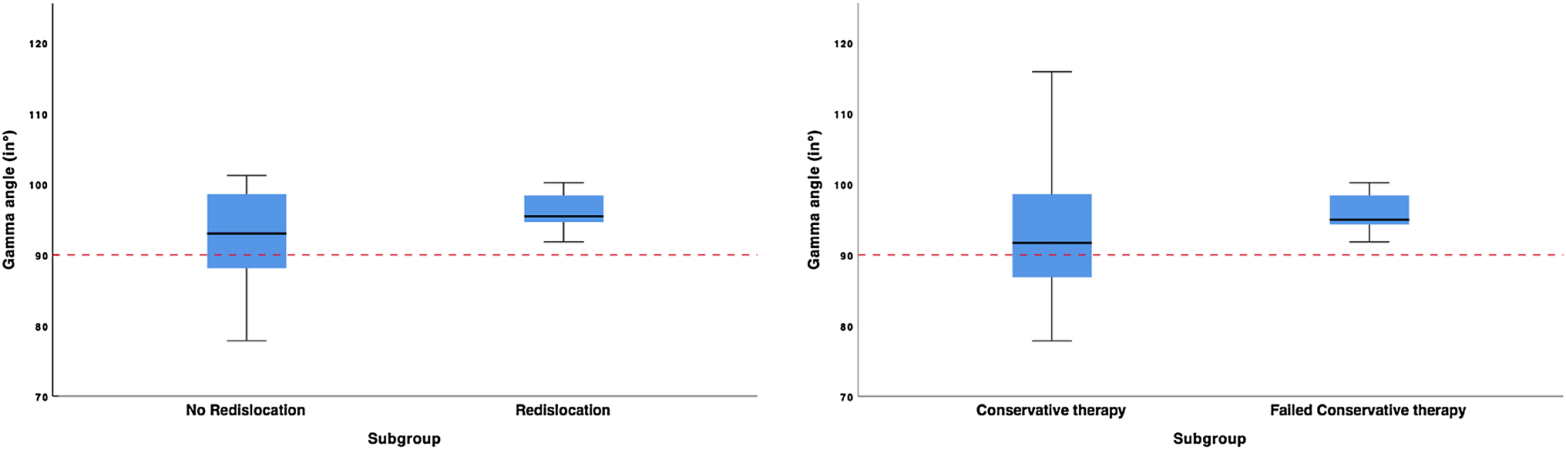
Subgroup corresponding adapted Gamma angle. Values of adapted gamma angle of all four subgroups. Dotted red lines present 90°

The adapted gamma angle was larger than 90° in all patients with failed conservative therapy (range, 91.9°–115.8°) and redislocations (range, 91.9°–100.2°), whereas it ranged from 77.8° to 115.9° in the no redislocation subgroup and from 77.8° to 115.9° in the conservative therapy group (Fig. 2).

The conservative subgroup older age at the time of dislocation showed a significant correlation with better clinical outcomes (SSV: *r* = 0.543, *p* = 0.02; ROWE: *r* = 0.418, *p* = 0.035; WOSI: *r* = 0.478, *p* = 0.045). A higher posterior glenohumeral subluxation correlated with a worse WOSI (*r* = − 0.590, *p* = 0.02) and follow-up posterior glenohumeral decentring (*r* = 0.68, *p* = 0.007) Furthermore, size and position (gamma angle) (*r* = 0.396, *p* = 0.039) and depth of the RHSL (*r* = 0.437, *p* = 0.023) correlated significantly with a worse grade of osteoarthritis of the joint at follow-up examination (Table 4).

**Table 4:** Association of patient and defect characteristics with clinical and radiological follow-up examination results in the conservative therapy subgroup (N = 20)

Characteristics	Outcomes				
	SSV correlation *p*-value	ROWE correlation *p*-value	WOSI correlation *p*-value	Follow-up glenohumeral centring correlation *p*-value	Follow-up osteoarthritis correlation *p*-value
Patient characteristics					
Age at dislocation	0.543 0.02	0.418 0.035	0.478 0.045	− 0.217 n.s.	0.277 n.s.
Follow-up	− 0.15 n.s.	− 0.129 n.s.	− 0.043 n.s.	− 0.28 n.s.	0.436 n.s.
Sex	0.192 n.s.	0.138 n.s.	0.057 n.s.	0.033 n.s.	0.116 n.s.
Affected side					
Right/left	0.182 n.s.	0.491 0.04	0.309 n.s.	0.137 n.s.	0.24 n.s.
Dominant/non-dominant	0.082 n.s.	0.213 n.s.	0.402 n.s.	0.234 n.s.	0.127 n.s.
Beighton score	0.095 n.s.	0.137 n.s.	0.004 n.s.	0.373 n.s.	− 0.211 n.s.
SSA score	0.436 n.s.	0.303 n.s.	0.51 n.s.	0.376 n.s.	0.240 n.s.
Posttraumatic radiological parameters					
Alpha angle	0.206 n.s.	0.279 n.s.	0.33 n.s.	− 0.102 n.s.	0.193 n.s.
Beta angle	0.001 n.s.	− 0.072 n.s.	0.181 n.s.	0.094 n.s.	0.254 n.s.
Gamma angle	0.129 n.s.	0.197 n.s.	0.374 n.s.	− 0.025 n.s.	0.396 0.039
Adapted gamma angle	0.139 n.s.	0.26 n.s.	0.379 n.s.	− 0.076 n.s.	0.664 0.004
Depth of RHSL	0.208 n.s.	0.01 n.s.	0.244 n.s.	0.004 n.s.	0.437 0.023
Glenoid defect area	− 0.142 n.s.	− 0.145 n.s.	0.028 n.s.	0.029 n.s.	0.079 n.s.
Glenoid defect diameter	− 0.149 n.s.	0.022 n.s.	− 0.099 n.s.	− 0.098 n.s.	0.07 n.s.
Glenohumeral centring	− 0.453 n.s.	− 0.237 n.s.	− 0.59 0.021	0.681 0.007	0.30 n.s.
Glenoid version	0.46 n.s.	0.108 n.s.	0.111 n.s.	− 0.50 n.s.	− 0.85 n.s.
Scapulohumeral centring	0.168 n.s.	0.18 n.s.	0.164 n.s.	0.539 0.038	− 0.044 n.s.

*SSV* subjective shoulder value, *WOSI* Western Ontario shoulder instability, *SSA-Score* subjective shoulder activity-score, *RHSL* reverse Hill–Sachs lesion

Considering the conservative subgroup, a comparison of the posttraumatic with the follow-up radiological characteristics of the RHSL revealed a statistically significant decrease in the depth of the defect (15.4 ± 5.7% posttraumatic vs. 11.7 ± 3.5% final follow-up, *p* = 0.007) while size and position did not show any difference (alpha angle 43.6° ± 8.7° vs. 42.6° ± 6.2°; beta angle 44.2° ± 11° vs. 48.3° ± 9.2°; gamma angle 87.9° ± 14° vs. 90.9° ± 10°).

## Discussion

The most important finding of the present study was that conservative treatment is a viable option in patients with an acute traumatic PSD with good clinical and radiological results after mid- to long -term follow-up. Posterior humeral head decentring, a higher gamma angle of the RHSL, and age were identified as relevant parameters associated with worse outcome.

In the literature, the success rate of conservative therapy in patients with PSI ranges between 8 and 70% [6, 7, 10, 12, 15] and patients with an atraumatic history of PSI tend to have more favourable outcomes with conservative management compared with those with a traumatic onset [34, 35]. However, the pathogenesis of posterior shoulder instability can be extremely variable and its determination is crucial in selecting the appropriate treatment [8]. Therefore, it is of uttermost importance to create homogenous cohorts of PSI when reporting outcome data using a classification system, such as the ABC classification used in this study. This classification distinguishes different groups of patients with posterior shoulder instability based on the pathomechanism and contains guiding principles on the necessary ensuing treatment [21]. A success rate of 69% of conservative therapy at mid- and long-term follow-up in patients with a traumatic acute PSD (Type A1 and A2 according to the ABC classification) was demonstrated in this study. However, some factors seem to be associated with inferior outcomes.

In a population-based study conducted in Olmsted County, Minnesota a high rate of secondary surgical intervention after initial conservative treatment for posterior shoulder instability in general was reported [34]. A trend towards higher BMI and more contact and weight-lifting activity was found in patients who had to undergo secondary surgery; however, the study did not differentiate between acute first-time posterior shoulder instability events and more chronic types of PSI. In addition, no analysis of structural defects as risk factors for failures was accomplished [34].

Acute traumatic posterior dislocations are frequently associated with impression fractures of the humeral head (HH), so called reverse Hill–Sachs lesions, which pose a risk of re-engagement of the HH with the posterior rim, leading to recurrent posterior instability [20]. The size of the RHSL, therefore, plays a key role in the determination of the necessity of surgical intervention [16, 25]. However, recent literature showed that not only the size but also the localisation of the RHSL needs to be considered to determine the risk of re-engagement and a standard combined measurement method for defect size and localisation, the so called gamma angle, was introduced [20]. Although a biomechanical study had calculated the critical gamma angle of approximately 90°, a further study showed that concomitant posterior glenoid bone defects might promote the engagement of noncritical RHSLs. This suggested the use of an adapted gamma angle by adding approximately 2° per millimetre posterior glenoid bone loss to the gamma angle measurement [19]. The present study confirms the biomechanically determined threshold of 90° gamma angle for conservative management of a reverse Hill–Sachs defect. All patients in the redislocation and failed conservative therapy group had an adapted gamma angle > 90°. On the other hand, about half of the patients with a successful conservative treatment had an adapted gamma angle > 90°, meaning that an adapted gamma angle > 90° does not necessarily require surgery. Nonetheless, a significant association between a higher adapted gamma angle and progression of osteoarthritis was shown in this study.

**Fig. 3:**
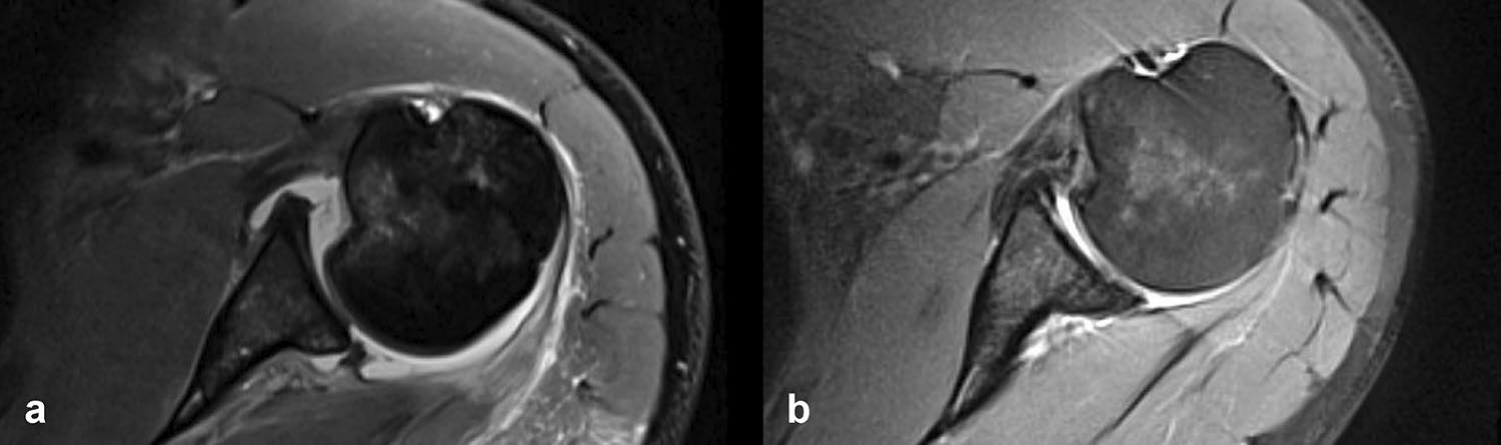
Morphological change in RHSL. Axial MRI images illustrating the change of the RHSL from posttraumatic imaging (**a**) to final follow-up imaging and (**b**) after 5 years of conservative treatment. A decrease in the depth of the defect and a consolidation of the posterior bony Bankart lesion can be seen; however, the posterior glenohumeral decentring apparently remained

This study also showed a significant association between depth of the RHSL with the osteoarthritic joint condition at follow-up. Interestingly, the depth of the defects decreased significantly from posttraumatic to final follow-up while size and position stayed the same. This observed decrease in the defect depth might be explained by consolidation of the fracture hematoma which fills the defect [17] (Fig. 3).

Posterior glenoid bone loss in the setting of posterior shoulder instability presents a rare and challenging situation which may lead to recurrent instability [2]. Although a posterior glenoid defect > 20% can lead to failure of arthroscopic soft tissue stabilisation and should be treated with a bony augmentation, there is no recommendation regarding the decision between conservative and operative treatment in case of a posterior bone loss lower than 20% [29]. While increasing bone loss may contribute to failure of conservative treatment in patients with traumatic acute PSD, our study failed to find a difference in glenoid bone loss between the study subgroups. This may be due to the low number and small amount of posterior glenoid defects observed. Similarly, a larger trial including 100 patients with PSI observed glenoid bone loss in 15% of the cases [23].

According to the results of this study, surgery should be considered in cases with posterior humeral subluxation as it is associated with a worse WOSI score at follow-up and a persisting static posterior glenohumeral head subluxation, which might lead to early-onset posterior decentring osteoarthritis[1, 31]. In addition, posttraumatic posterior glenohumeral subluxation was much higher in the redislocation group compared to the no redislocation group without reaching statistical significance, thus warranting further studies (Fig. 3).

**Fig. 4:**
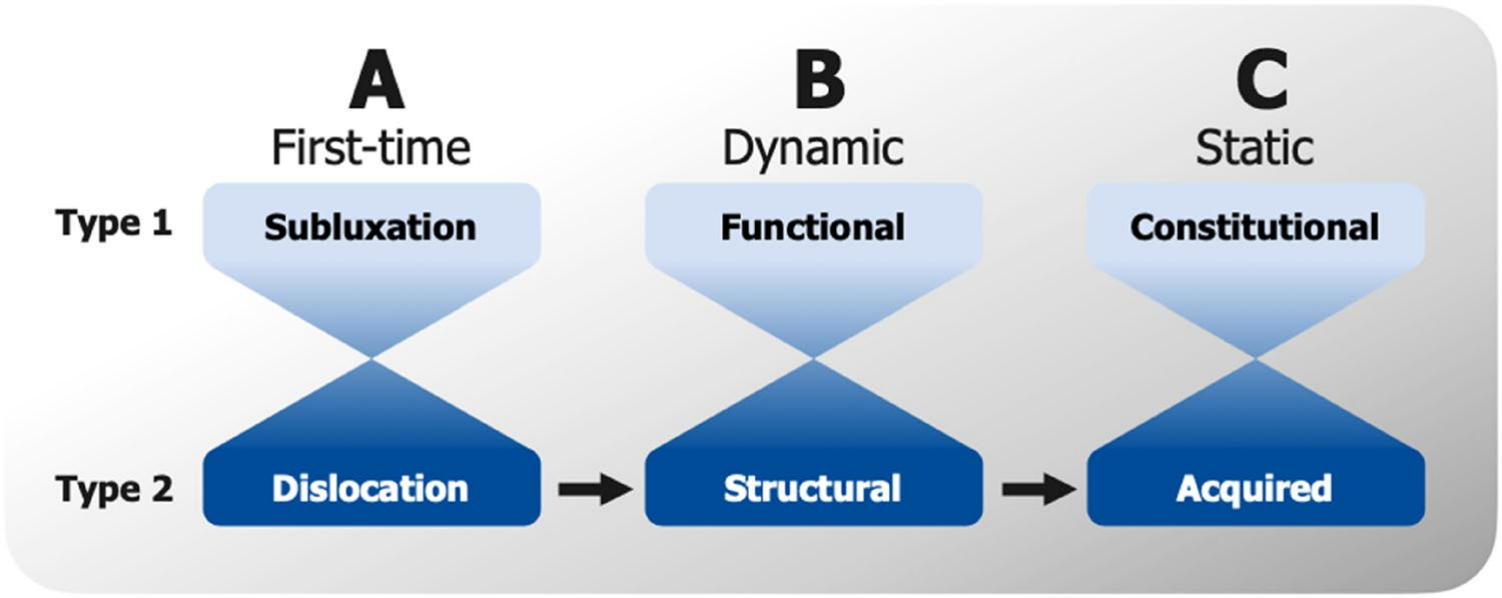
ABC classification. The ABC classification of posterior shoulder instability according to Moroder et al. [21]. There is a gradual transition from type 1 to type 2 and vice versa as well as the possibility of progression from type A2 to Type B2 to Type C2

In general, the findings of this study confirm the theory that a patient can progress from acute posterior shoulder dislocation (A2) to structural dynamic posterior shoulder instability (B2) and acquired static posterior shoulder instability (C2) according to the ABC classification [21] (Fig. 4).

Similar to the findings in anterior shoulder instability [24], the age at initial dislocation seems to have a high impact on the clinical outcome in patients with acute traumatic PSD. While younger age was associated with a worse clinical outcome, higher age was associated with better clinical scores at the last follow-up. This might be explained by age-related lower shoulder-specific demands.

A limitation of this study is the retrospective collection of data. However, pre-interventional clinical scores are not required in these acute trauma cases and only patients with available CT or MRI scans after trauma which could be retrospectively assessed were included. All patients were invited for follow-up and evaluated clinically. However, there was a rather high rate of loss to follow-up which might be explained by the fact that the minimum follow-up period was quite long. Nonetheless, to our knowledge, this is the first study assessing mid- to long-term clinical results of conservative treatment in a homogeneous cohort of patients with acute PSDs Type A1 and A2. The rather small sample may be discussed as to underpower the study for detecting certain risk factors for inferior outcome in the subgroup analysis. However, relevant associations were identified which might help clinical decision making in the future. Assessment of age, glenohumeral subluxation, and the gamma angle is recommended to decide between conservative and surgical treatment of patients with acute posterior shoulder dislocation Type A.

## Conclusion

Conservative treatment is a viable option in patients with an acute traumatic posterior shoulder dislocation with good outcome after mid- and long-term follow-up especially in patients with centred joint, low gamma angle, and middle or old age.

The findings of this study confirm the theory that a patient can progress from acute posterior shoulder dislocation (A2) to structural dynamic posterior shoulder instability (B2) and or acquired static posterior shoulder instability (C2).

## Abbreviations

AGA: Arbeitsgemeinschaft für Arthroskopie und Gelenkchirurgie
CI: Confidence interval
CIA: Collective instability arthropathy
CT: Computerised tomography
ICC: Inter- and intraclass correlation coefficient
MRI: Magnetic resonance imaging
PSD: Posterior shoulder dislocation
PSI: Posterior shoulder instability
RHSL: Reverse Hill–Sachs lesion
SSV: Subjective shoulder value
SSAS: Shoulder sports activity score
WOSI: Western Ontario shoulder instability

## References

[1] Akgün D, Siegert P, Danzinger V, Plachel F, Minkus M, Thiele K (2021). Glenoid vault and humeral head alignment in relation to the scapular blade axis in young patients with pre-osteoarthritic static posterior subluxation of the humeral head. J Shoulder Elbow Surg.

[2] Antosh IJ, Tokish JM, Owens BD (2016). Posterior shoulder instability. Sports Health.

[3] Baudi P, Righi P, Bolognesi D, Rivetta S, Rossi Urtoler E, Guicciardi N (2005). How to identify and calculate glenoid bone deficit. Chir Organ Mov.

[4] Beighton P, Solomon L, Soskolne CL (1973). Articular mobility in an African population. Ann Rheum Dis.

[5] Bois AJ, Fening SD, Polster J, Jones MH, Miniaci A (2012). Quantifying glenoid bone loss in anterior shoulder instability: reliability and accuracy of 2-dimensional and 3-dimensional computed tomography measurement techniques. Am J Sports Med.

[6] Burkhead WZ, Rockwood CA (1992). Treatment of instability of the shoulder with an exercise program. J Bone Joint Surg Am.

[7] Christensen DL, Elsenbeck MJ, Wolfe JA, Nickel WN, Roach W, Waltz RA (2020). Risk factors for failure of nonoperative treatment of posterior shoulder labral tears on magnetic resonance imaging. Mil Med.

[8] Frank RM, Romeo AA, Provencher MT (2017). Posterior glenohumeral instability: evidence-based treatment. J Am Acad Orthop Surg.

[9] Friedman RJ, Hawthorne KB, Genez BM (1992). The use of computerized tomography in the measurement of glenoid version. J Bone Joint Surg Am.

[10] Fronek J, Warren RF, Bowen M (1989). Posterior subluxation of the glenohumeral joint. J Bone Joint Surg Am.

[11] Gilbart MK, Gerber C (2007). Comparison of the subjective shoulder value and the Constant score. J Shoulder Elbow Surg.

[12] Hurley JA, Anderson TE, Dear W, Andrish JT, Bergfeld JA, Weiker GG (1992). Posterior shoulder instability. Surgical versus conservative results with evaluation of glenoid version. Am J Sports Med.

[13] Kidder JF, Rouleau DM, Pons-Villanueva J, Dynamidis S, DeFranco MJ, Walch G (2010). Humeral head posterior subluxation on CT scan: validation and comparison of 2 methods of measurement. Tech Shoulder Elb Surg.

[14] Kirkley A, Griffin S, McLintock H, Ng L (1998). The development and evaluation of a disease-specific quality of life measurement tool for shoulder instability. The Western Ontario Shoulder Instability Index (WOSI). Am J Sports Med.

[15] Lee J, Woodmass JM, Bernard CD, Leland DP, Keyt LK, Krych AJ et al (2021) Nonoperative management of posterior shoulder instability: what are the long-term clinical outcomes? Clin J Sport Med. 10.1097/jsm.0000000000000907 10.1097/JSM.0000000000000907 33852434

[16] Longo UG, Ciuffreda M, Locher J, Casciaro C, Mannering N, Maffulli N (2020). Posterior shoulder instability: a systematic review. Br Med Bull.

[17] Marsell R, Einhorn TA (2011). The biology of fracture healing. Injury.

[18] Moroder P, Odorizzi M, Pizzinini S, Demetz E, Resch H, Moroder P (2015). Open Bankart repair for the treatment of anterior shoulder instability without substantial osseous glenoid defects: results after a minimum follow-up of twenty years. J Bone Joint Surg Am.

[19] Moroder P, Plachel F, Tauber M, Habermeyer P, Imhoff A, Liem D (2017). Risk of engagement of bipolar bone defects in posterior shoulder instability. Am J Sports Med.

[20] Moroder P, Runer A, Kraemer M, Fierlbeck J, Niederberger A, Cotofana S (2015). Influence of defect size and localization on the engagement of reverse Hill-Sachs lesions. Am J Sports Med.

[21] Moroder P, Scheibel M (2017). ABC classification of posterior shoulder instability. Obere Extremität.

[22] Moroder P, Tauber M, Hoffelner T, Auffarth A, Korn G, Bogner R (2013). Reliability of a new standardized measurement technique for reverse Hill-Sachs lesions in posterior shoulder dislocations. Arthroscopy.

[23] Moroder P, Tauber M, Scheibel M, Habermeyer P, Imhoff AB, Liem D (2016). Defect characteristics of reverse Hill-Sachs lesions. Am J Sports Med.

[24] Olds M, Ellis R, Donaldson K, Parmar P, Kersten P (2015). Risk factors which predispose first-time traumatic anterior shoulder dislocations to recurrent instability in adults: a systematic review and meta-analysis. Br J Sports Med.

[25] Paul J, Buchmann S, Beitzel K, Solovyova O, Imhoff AB (2011). Posterior shoulder dislocation: systematic review and treatment algorithm. Arthroscopy.

[26] Portney LG (2020) Foundations of clinical research: applications to evidence-based practice. FA Davis

[27] Rowe CR, Patel D, Southmayd WW (1978). The Bankart procedure: a long-term end-result study. J Bone Joint Surg Am.

[28] Samilson RL, Prieto V (1983). Dislocation arthropathy of the shoulder. J Bone Joint Surg Am.

[29] Schwartz DG, Goebel S, Piper K, Kordasiewicz B, Boyle S, Lafosse L (2013). Arthroscopic posterior bone block augmentation in posterior shoulder instability. J Shoulder Elbow Surg.

[30] Stein T, Linke RD, Buckup J, Efe T, von Eisenhart-Rothe R, Hoffmann R (2011). Shoulder sport-specific impairments after arthroscopic Bankart repair: a prospective longitudinal assessment. Am J Sports Med.

[31] Walch G, Ascani C, Boulahia A, Nové-Josserand L, Edwards TB (2002). Static posterior subluxation of the humeral head: an unrecognized entity responsible for glenohumeral osteoarthritis in the young adult. J Shoulder Elbow Surg.

[32] Walch G, Badet R, Boulahia A, Khoury A (1999). Morphologic study of the glenoid in primary glenohumeral osteoarthritis. J Arthroplasty.

[33] Watson L, Balster S, Warby SA, Sadi J, Hoy G, Pizzari T (2017). A comprehensive rehabilitation program for posterior instability of the shoulder. J Hand Ther.

[34] Woodmass JM, Lee J, Johnson NR, Wu IT, Camp CL, Dahm DL (2019). Nonoperative management of posterior shoulder instability: an assessment of survival and predictors for conversion to surgery at 1 to 10 years after diagnosis. Arthroscopy.

[35] Wooten CJ, Krych AJ, Schleck CD, Hudgens JL, May JH, Dahm DL (2015). Arthroscopic capsulolabral reconstruction for posterior shoulder instability in patients 18 years old or younger. J Pediatr Orthop.

